# Differential Expression Study of Lysine Crotonylation and Proteome for Chronic Obstructive Pulmonary Disease Combined with Type II Respiratory Failure

**DOI:** 10.1155/2021/6652297

**Published:** 2021-06-15

**Authors:** Qing Gan, Donge Tang, Qiang Yan, Jiejing Chen, Yong Xu, Wen Xue, Lu Xiao, Fengping Zheng, Huixuan Xu, Yingyun Fu, Yong Dai

**Affiliations:** ^1^Guangxi Key Laboratory of Metabolic Disease Research, Department of Clinical Laboratory of Guilin No. 924 Hospital, Guilin 541002, Guangxi, China; ^2^Clinical Medical Research Center, The Second Clinical Medical College of Jinan University, Shenzhen People's Hospital, Shenzhen, Guangdong 518020, China; ^3^Organ Transplantation Center of Guilin No. 924 Hospital, Guilin 541002, Guangxi, China; ^4^Key Laboratory of Shenzhen Respiratory Diseases, Department of Pulmonary and Critical Care Medicine, Shenzhen Institute of Respiratory Disease, The First Affiliated Hospital of Southern University of Science and Technology, The Second Clinical Medical College, Jinan University, Shenzhen People's Hospital, Shenzhen 518020, China

## Abstract

**Introduction:**

The modification of lysine crotonylation (Kcr) is another biological function of histone in addition to modification of lysine acetylation (Kac), which may play a specific regulatory role in diseases.

**Objectives:**

This study compared the expression levels of Kcr and proteome between patients with chronic obstructive pulmonary disease (COPD) combined with type II respiratory failure (RF) to study the relationship between Kcr, proteome, and COPD.

**Methods:**

We tested the Kcr and proteome of COPD combined with type II RF and normal control (NC) using croton acylation enrichment technology and liquid chromatography tandem mass spectrometry (LC-MS/MS) with high resolution.

**Results:**

We found that 32 sites of 23 proteins were upregulated and 914 sites of 295 proteins were downregulated. We performed Kyoto Encyclopedia of Genes and Genomes (KEGG), protein domain, and Gene Ontology (GO) analysis on crotonylated protein. In proteomics research, we found that 190 proteins were upregulated and 151 proteins were downregulated. Among them, 90 proteins were both modified by differentially expressed crotonylation sites and differentially expressed in COPD combined with type II RF and NC.

**Conclusion:**

Differentially expressed crotonylation sites may be involved in the development of COPD combined with type II RF. 90 proteins modified by crotonylation and differentially expressed in COPD combined with type II RF can be used as markers for the study of the molecular pathogenesis of COPD combined with type II RF.

## 1. Introduction

Chronic obstructive pulmonary disease (COPD) is a common respiratory disease caused by incomplete reversible and progressive development of airflow limitation [1]. The main causes of COPD are inhalation of smoke, sensitization of respiratory tract, occupational exposure, and air pollution [2]. Respiratory failure (RF) is a condition in which the respiratory system fails in one or both of its gas exchange functions. The cause is hypoxemia caused by lung failure or hypoxemia caused by pump failure or alveolar hypoventilation and hypercapnia caused by pump failure [3]. RF is divided into type I and type II. RF is a serious complication of COPD, and the main cause of death from COPD is type II RF. Our national statistics of research found that patients with COPD combined with type II are older than type I. At present, the clinical treatment methods for COPD patients mainly include smoking cessation, drugs, exercise rehabilitation, mechanical ventilation, and lung volume reduction surgery. However, due to delayed patient visits, the rate of pulmonary function tests is low, and patients often do not receive timely and effective treatment. Therefore, early diagnosis and treatment of COPD combined with type II RF is of great significance to patients and families.

Lysine acetylation (Kac) is the earliest and most studied type of modification [4]. The lysine crotonylation (Kcr) is a newly identified biological function of posttranslational modification, which mainly occurs in the lysine residues of histones [5]. The Kcr is similar to the Kac modification in the structure, the regulatory enzyme system, and the recognition protein. However, the crotonylation modification is more potent for gene expression than the acetylation modification, and the balance of histone crotonylation modification and acetylation modification has an effect on gene expression [6]. At present, studies have confirmed that Kcr has the functions of regulating gene transcription, participating in sperm formation and stress protection of acute kidney injury [6–10]. Montellier et al. found that the Kcr modification had a high level in the round sperm chromosome, and the Kcr modification sites were abundantly present at the transcription initiation site, thereby promoting the expression levels of the gene [11]. Xu et al. found that crotonylated modified nonhistones in H1299 cells may be involved in multiple signaling pathways and cellular functions, such as involvement in transport, formation of ribosomes, and Parkinson's disease pathways [12]. Recently, a high degree of agreement between crotonylated proteins and crotonylation sites in zebrafish embryos and humans was reported, and Kcr regulates muscle contraction and protein synthesis [13]. Therefore, Kcr may have an effect on many other diseases and may be a novel class of biomarkers for diagnosis, evaluation, and treatment targets. This study hopes to identify some Kcr of new and reliable biomarkers, so as to provide a data basis for the clinical research of COPD combined with type II RF.

## 2. Materials and Methods

### 2.1. Samples Collection

Peripheral blood samples were collected from 8 patients with COPD combined with type II RF and 36 healthy individuals from the Shenzhen People's Hospital (Shenzhen, China). 36 healthy individuals belonged to the normal controls (NC) in this study. All 8 patients (5 males and 3 females; mean age, 75.75 ± 12.02) had long-term symptoms of cough, shortness of breath, and lung infection. The mean value of PaCO_2_ (mmHg) in blood gas analysis is 52.96 ± 22.63, and PaO_2_ (mmHg) in blood gas analysis is 80.70 ± 24.78. PaCO_2_ (mmHg) > 50 mmHg and PaO2 (mmHg) < 60 mmHg were considered type II RF. Among them, 5 males have mean pack-years of smoking 20.00 ± 7.07 years and quit smoking. They did not have severe liver and kidney disease and family genetic history and had not received hormone therapy recently. The clinical characteristics of patients are summarized in Table 1. All of the peripheral blood samples were obtained after receiving informed consent from the participating subject. And the peripheral blood was obtained when the patients were admitted to the hospital because of the deterioration of the condition. This study was performed according to the guidelines of the Shenzhen People's Hospital, which abided by the Helsinki Declaration on ethical principles for medical research involving human subjects. Then, peripheral blood mononuclear cells (PBMCs) were isolated by density gradient centrifugation using Ficoll-Hypaque (GE Healthcare Bio-Sciences AB, Uppsala, Sweden). PBMCs were lysed with TRIzol reagent (Invitrogen, Carlsbad, CA) and stored at −80°C.

### 2.2. Quantitative Analysis of Kcr and Proteomics

#### 2.2.1. Protein Extraction

Samples were removed from −80°C and added to 4 volumes of lysis buffer (8 M urea, 1% protease inhibitor cocktail, 3 *μ*M TSA, 50 mM NAM, and 2 mM EDTA) for sonication. After centrifugation at 12,000 g for 10 min at 4°C, the cell debris was removed, the supernatant was transferred to a new centrifuge tube, and the protein concentration was determined using a BCA kit.

#### 2.2.2. Trypsin Digestion

Dithiothreitol was added to the protein solution to a final concentration of 5 mM and reduced at 56°C for 30 min. Iodoacetamide was then added to a final concentration of 11 mM and incubated for 15 min at room temperature in the dark. Finally, the urea concentration of the sample is diluted to less than 2 M. Trypsin was added at a mass ratio of 1 : 50 (pancreatin: protein), digested overnight at 37 °C, added at a mass ratio of 1 : 100 (pancreatin: protein), and continued to digest for 4 h.

#### 2.2.3. Relative and Absolute Quantitation (TMT/iTRAQ) Labeling

The trypsin-digested peptide was desalted with Strata X C18 (Phenomenex) and vacuum-dried. The peptide was solubilized with 0.5 M TEAB and the peptide was labeled according to the TMT kit instructions.

#### 2.2.4. High-Performance Liquid Chromatography (HPLC) Fractionation

For proteome experiment, the tryptic peptides were fractionated into fractions by high pH reverse-phase HPLC using Agilent 300Extend C18 column (5 *μ*m particles, 4.6 mm ID, and 250 mm length).

#### 2.2.5. The Kcr Modification Enrichment

The peptides were dissolved in IP buffer solution (100 mM NaCl, 1 mM EDTA, 50 mM Tris-HCl, 0.5% NP-40, and pH 8.0), and the supernatant was transferred to the prewashed crotonylated resin (PTM503, from Hangzhou Jingjie Biotechnology Co., Ltd., PTM Bio) and placed on a rotary shaker at 4 °C. The resin was washed 4 times with IP buffer solution and twice with deionized water after incubation. At that time, the 0.1% trifluoroacetic acid eluent eluted the resin-bound peptide three times. Finally, the eluate was collected and dried in vacuo. After draining, the salt of peptides was removed according to C18 ZipTips instructions for liquid chromatography tandem mass spectrometry (LC-MS/MS) analysis.

#### 2.2.6. LC-MS/MS Analysis

The peptide was dissolved in liquid phase A phase (0.1% aqueous formic acid) and separated using an EASY-nLC 1000 ultrahigh-performance liquid chromatography (UPLC) system. The gradient was comprised of solvent B. Liquid phase gradient setting was 0∼38 min, 8%–22% solvent B; 38∼52 min, 22%–35% solvent B; 52∼56 min, 35%–80% solvent B; 56∼60 min, 80% solvent B.

The peptides were dissociated by a UPLC system and injected into an NSI ion source for ionization and analyzed by Q Exactive™ Plus (Thermo).

#### 2.2.7. Database Search

Secondary mass spectral data was retrieved using MaxQuant (v1.5.2.8).

For searching Kcr database, the effect was set to trypsin/P (the number of missed sites is set to 4). The minimum length of the peptide is set to 7 amino acid residues, and the maximum number of modifications of the peptide is set to 5. The mass tolerance for precursor ions of first search and main search was set to 20 ppm and 5 ppm, respectively, and the mass error tolerance of the secondary fragment ions was 0.02 Da. Carbamidomethyl on Cys was designated as fixed modification. Acetylation modification, Kcr modification, and oxidation on Met were designated as variable modifications. FDR was adjusted to <1% and the minimum score for modified peptides was set to >40.

For searching proteome database, the effect was set to trypsin/P (the number of missed sites is set to 2). The minimum length of the peptide is set to 7 amino acid residues, and the maximum number of modifications of the peptide is set to 5. The mass tolerance for precursor ions of first search and main search was set to 20 ppm and 5 ppm, respectively, and the mass error tolerance of the secondary fragment ions was 0.02 Da. Carbamidomethyl on Cys was designated as fixed modification and oxidation on Met was designated as variable modifications. FDR was adjusted to <1% and the minimum score for peptides was set to >40.

#### 2.2.8. Gene Ontology (GO) Annotation

GO annotation proteome was derived from the UniProt-GOA database (www. http://www.ebi.ac.uk/GOA/). Firstly, convert identified protein ID to UniProt ID and then map it to GO IDs by protein ID. Then proteins were classified by GO annotation based on three categories: biological process, cellular component, and molecular function. GO with a corrected value of *P* < 0.05 was considered significant.

#### 2.2.9. Domain Annotation

The protein domain annotations were performed on the identified proteins using the protein sequence algorithm-based software InterProScan and the corresponding InterPro domain database. The InterPro domain database (http://www.ebi.ac.uk/interpro/) is a free web database that integrates information including protein family classification, protein domain classification, and protein functional site classification. Protein domains with a corrected value of *P* < 0.05 were considered significant.

#### 2.2.10. Kyoto Encyclopedia of Genes and Genomes (KEGG) Pathway Annotation

We used the KEGG pathway database to annotate the protein pathway. First, the submitted protein was annotated using the KEGG online service tool KAAS (http://www.genome.jp/tools/kaas/), and the annotated protein was then matched into the corresponding pathway in the database by the KEGG mapper. The pathway with a corrected value of *P* < 0.05 was considered significant. These pathways were classified into hierarchical categories according to KEGG website.

#### 2.2.11. Subcellular Localization

We used WoLF PSORT, a subcellular localization prediction software, to predict subcellular localization.

## 3. Result

### 3.1. Quantitative Analysis of Kcr Modification in COPD and NC

A total of 946 crotonylation sites containing quantitative information of differential expression were identified by LC-MS/MS in 318 proteins between COPD combined with type II RF and NC The fold change was more than 1.2 times as a significant upregulation and less than 1/1.2 as a criterion for significant downregulation. Among them, 32 sites of 23 proteins were upregulated and 914 sites of 295 proteins were downregulated. The quality control test standard for mass spectrometry (MS) data is that the mass error is concentrated below 0 and 10 ppm, indicating that the mass accuracy of the MS data meets the requirements. Most peptides ranged in length from 8 to 20 amino acids, which were consistent with the rule of trypsin-digesting peptides, indicating that sample preparation is up to standard. The quality control of the peptides of all samples in this study reached the standard.

### 3.2. GO Functional Annotation and Subcellular Localization of Crotonylation Modification Sites Corresponding to Proteins

**Figure 1:**
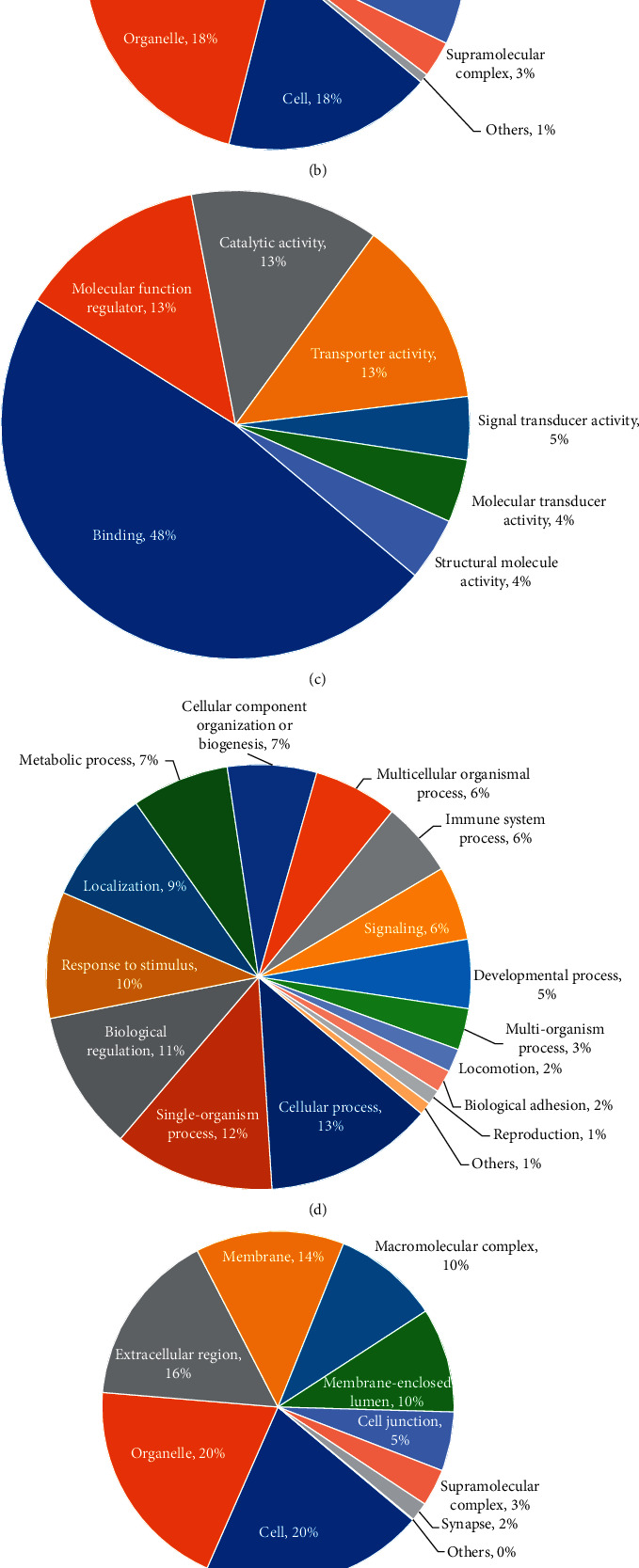
GO functional annotation of crotonylated proteins. GO analysis of upregulated crotonylated proteins shows biological processes (a), cellular component (b), and molecular function (c). GO analysis of downregulated crotonylated proteins shows biological processes (d), cellular component (e), and molecular function (f).

We performed a GO functional classification of biological processes, cellular components, and molecular functions of quantitative proteins. In upregulated crotonylated proteins, most crotonylated proteins were involved in response to stimulus, single-organism process, organelle, cell, and binding, accounting for 11%, 11%, 18%, 18%, and 48% of all crotonylated proteins, respectively (Figures 1(a)–1(c)). In downregulated crotonylated proteins, most crotonylated proteins were involved in cellular process, single-organism process, cell, organelle, extracellular region, binding, and catalytic activity, accounting for 13%, 12%, 20%, 20%, 16%, 52%, and 23% of all crotonylated proteins, respectively (Figures 1(d)–1(f)). The upregulated crotonylated proteins and downregulated crotonylated proteins most were involved in organelle, cell, and binding.

**Figure 2:**
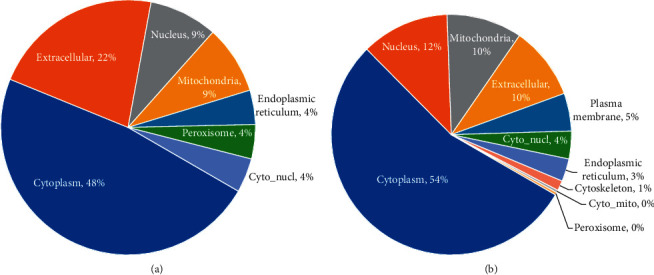
Subcellular localization of upregulated (a) and downregulated (b) crotonylated proteins.

We used WoLF PSORT to predict the subcellular localization of crotonylated proteins. Most upregulated crotonylated proteins were distributed in the cytoplasm (48%) and extracellular (22%). Most downregulated proteins were distributed in the cytoplasm (54%), nucleus (12%), and mitochondria (10%) (Figures 2(a) and 2(b)). The upregulated crotonylated proteins and downregulated crotonylated proteins most were distributed in the cytoplasm.

### 3.3. Functional Enrichment Analysis of Crotonylation Modification Sites Corresponding to Proteins

In order to detect whether the differential expression has a significant enrichment trend in some functional types, we performed enrichment analysis of functional annotation types such as GO, KEGG, and protein domains for the corresponding proteins of crotonylation modification sites. The *P*-value obtained by the enrichment test (Fisher's exact test) is converted by negative logarithm (−log10). The larger the converted value is, the more significant the richness of this functional type is.

**Figure 3:**
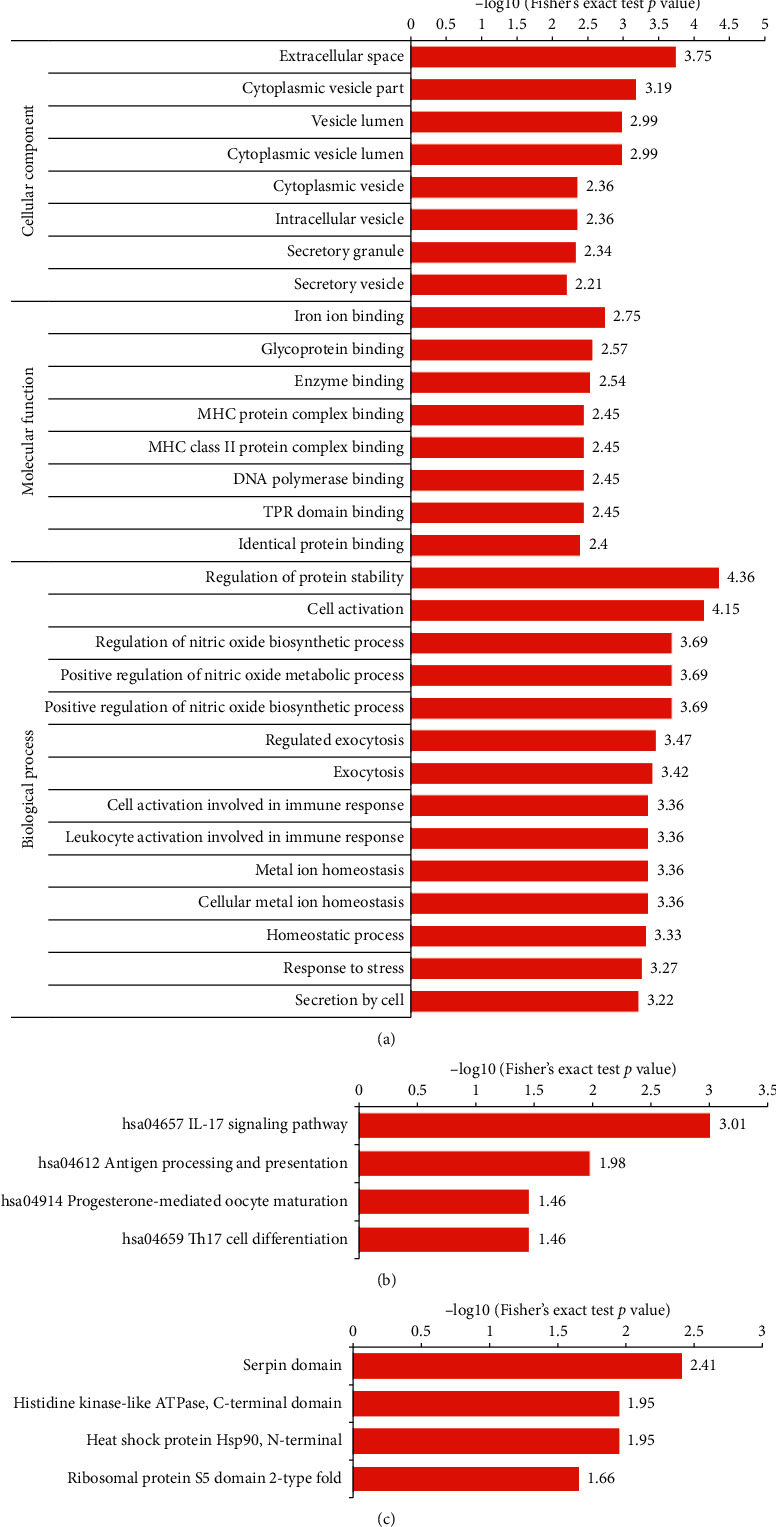
Functional enrichment analysis of crotonylation modification sites corresponding to proteins. (a) Enrichment of GO analysis of upregulated crotonylated protein. (b) Enrichment of KEGG pathway analysis of upregulated crotonylated protein. (c) Enrichment of protein domain analysis of upregulated crotonylated protein.

The enrichment analysis of the cellular components revealed that the upregulated crotonylated proteins were highly enriched in the extracellular space, cytoplasmic vesicle part, vesicle lumen, and cytoplasmic vesicle lumen. The enrichment results of the molecular function category showed that the upregulated crotonylated proteins were highly enriched in the iron ion binding, glycoprotein binding, and enzyme binding. The enrichment results of the biological processes showed that the upregulated crotonylated proteins were highly enriched in the regulation of protein stability, cell activation, regulation of nitric oxide biosynthetic process, positive regulation of nitric oxide metabolic process, and positive regulation of nitric oxide biosynthetic process (Figure 3(a)). The KEGG pathway enrichment analysis showed that the upregulated crotonylated proteins were mainly enriched in the hsa04657 IL-17 signaling pathway and hsa04612 antigen processing and presentation (Figure 3(b)). The upregulated crotonylated protein domains were significantly enriched in the serpin domain, histidine kinase-like ATPase, C-terminal domain, heat shock protein Hsp90, N-terminal, and ribosomal protein S5 domain 2-type fold (Figure 3(c)). It is suggested that the Kcr plays an important role in these processes. However, there is no downregulation of crotonylation protein enrichment in GO analysis, KEGG pathway, and protein domains.

### 3.4. Interaction between Differential Expression of Kcr and Proteins

In this project experiment, 341 proteins' quantitative information was obtained by LC-MS/MS between COPD combined with type II RF and NC. The fold change was more than 1.2 times as a significant upregulation and less than 1/1.2 as a criterion for significant downregulation. Among them, 190 proteins were upregulated and 151 proteins were downregulated.

**Figure 4:**
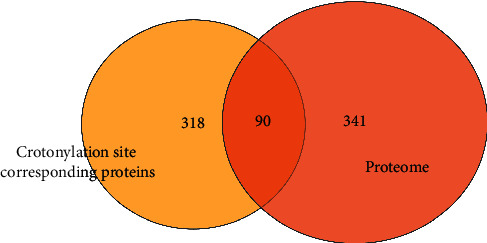
Common proteins of differentially expressed crotonylation site corresponding proteins and proteome between COPD and NC.

We analyzed the expression profiles of differentially expressed crotonylation site corresponding protein and proteome and conducted a correlation analysis between Kcr and proteome. As a result, there were 90 identical proteins in the crotonylated proteins and proteome with quantitative information. This result showed that these 90 proteins were both modified by differentially expressed crotonylation sites and differentially expressed in COPD combined with type II RF and NC (Figure 4 and Table 2).

## 4. Discussion

Since COPD has been defined as a heterogeneous disease [14], more and more researchers have conducted molecular biology research, including genomics [15–19] and metabolomics [20, 21]. For proteomics, Braido et al. [22] found that there is a possible link between the innate immune system and the worsening of COPD infectivity by discovering that the immune-associated Clara cell 16 (CC-16) is differentially expressed in COPD. Leuzzi et al. [23] found that high baseline C-reactive protein (CRP) levels are significantly associated with late mortality in COPD patients. Recently, Xiang et al. [24] found that YKL-40 proteins were differentially expressed in COPD, suggesting that YKL-40 may be involved in bronchial inflammation and remodeling of COPD and may be a useful biomarker for the diagnosis and monitoring of COPD. Although the relationship between proteomics and COPD has been the focus of research, the relationship between Kcr and COPD combined with type II RF is largely unknown.

In the GO enrichment, the upregulated crotonylated proteins were enriched in biological processes, cell structural components, and cell-binding functional types, indicating that Kcr participated in pathologically related cell and molecular life activities in COPD combined with type II RF. The functional enrichment of Kcr in KEGG analysis revealed IL-17 signaling pathway, antigen processing, and presentation, all of which have an association with immunology [25, 26]. Mario and Maria [27] have also shown that IL-17 plays an important proinflammatory effect in COPD. The lung infection of COPD is mainly in the large and small airway position, with bronchial mucosal edema, inflammation, and hypersecretion of gland as the main clinical manifestations [28]. It is suggested that the differentially expressed Kcr sites enriched in IL-17 signaling pathway and antigen processing and presentation may regulate the expression of related proteins in the immune response of COPD lung infection.

We used TMT labeling, crotonylation enrichment technology, and the quantitative proteomics research strategy of high-resolution LC-MS/MS to quantitatively study Kcr and proteomics in COPD combined with type II RF and NC. We found 32 and 914 sites, corresponding to 23 and 295 proteins, which were significantly upregulated and downregulated, respectively. We also conducted differential expression studies on proteomics. We found that 90 proteins were both modified by differentially expressed crotonylation sites and differentially expressed in COPD and NC. These 90 proteins were differentially expressed between COPD combined with type II RF and NC and may be biomarkers for the diagnosis of COPD combined with type II RF.

The present study has some limitations. Firstly, the small number of samples may cause certain errors in the research results. Secondly, this research provides a data basis for the correlation between Kcr and COPD combined with type II RF, which is only the initial stage. Therefore, we will consider collecting more samples for subsequent research. In a later study, we will consider performing site-directed mutagenesis in vitro and introduce site mutation for proteins and sites that modify levels of difference. Immunofluorescence (IF), fluorescent resonance energy transfer (FRET), and other fluorescent labeling methods will be used to analyze the subcellular localization and real-time dynamic changes of the target molecule, which will provide a basis for functional mechanism research and protein interaction.

## 5. Conclusion

We found that differentially expressed Kcr may be involved in the modification of COPD-related proteins and is involved in the pathogenesis of COPD combined with type II RF. Our research provides an idea for studying the association of Kcr, proteins, and COPD to identify biomarkers of COPD combined with type II RF.

## Figures and Tables

**Table 1:** Detail clinical information for all COPD combined with type II RF.

No.	Gender	Age	PaCO_2_ (mmHg)	PaO_2_ (mmHg)	Smoke (pack-years)
P1	Male	79	64.3	57.1	20
P2	Male	66	89.9	127.0	30
P3	Male	50	30.0	74.8	10
P4	Male	82	49.0	74.7	20
P5	Male	87	33.5	82.0	20
P6	Female	79	28.9	108.0	—
P7	Female	82	77.2	62.0	—
P8	Female	81	50.9	60.0	—

**Table 2:** 90 protein expression fold change value.

Protein description	Protein fold change	Protein-regulated type
FYN-binding protein OS = *Homo sapiens* GN = FYB	0.788	Down
Flotillin-1 OS = *Homo sapiens* GN = FLOT1	1.452	Up
Haptoglobin OS = *Homo sapiens* GN = HP	0.462	Down
Carbonic anhydrase 1 OS = *Homo sapiens* GN = CA1	2.024	Up
Carbonic anhydrase 2 OS = *Homo sapiens* GN = CA2	1.39	Up
Alpha-1-antitrypsin OS = *Homo sapiens* GN = SERPINA1	0.475	Down
Ig kappa chain C region OS = *Homo sapiens* GN = IGKC	0.411	Down
Ig gamma-1 chain C region OS = *Homo sapiens* GN = IGHG1	0.386	Down
Ig gamma-2 chain C region OS = *Homo sapiens* GN = IGHG2	0.468	Down
“Spectrin alpha chain, erythrocytic 1 OS = *Homo sapiens* GN = SPTA1”	1.522	Up
Apolipoprotein A-I OS = *Homo sapiens* GN = APOA1	0.299	Down
Apolipoprotein A-II OS = *Homo sapiens* GN = APOA2	0.197	Down
Fibrinogen alpha chain OS = *Homo sapiens* GN = FGA	0.803	Down
Fibrinogen beta chain OS = *Homo sapiens* GN = FGB	0.819	Down
Fibrinogen gamma chain OS = *Homo sapiens* GN = FGG	0.791	Down
Platelet basic protein OS = *Homo sapiens* GN = PPBP	0.715	Down
Serotransferrin OS = *Homo sapiens* GN = TF	0.271	Down
Lactotransferrin OS = *Homo sapiens* GN = LTF	1.463	Up
Catalase OS = *Homo sapiens* GN = CAT	1.416	Up
Apolipoprotein B-100 OS = *Homo sapiens* GN = APOB	0.668	Down
Cytochrome b-245 heavy chain OS = *Homo sapiens* GN = CYBB	1.361	Up
Protein S100-A8 OS = *Homo sapiens* GN = S100A8	1.42	Up
Myeloperoxidase OS = *Homo sapiens* GN = MPO	1.348	Up
Glucose-6-phosphate isomerase OS = *Homo sapiens* GN = GPI	1.307	Up
Protein disulfide-isomerase OS = *Homo sapiens* GN = P4HB	1.531	Up
Annexin A2 OS = *Homo sapiens* GN = ANXA2	0.792	Down
Annexin A6 OS = *Homo sapiens* GN = ANXA6	0.782	Down
Heat shock protein HSP 90-beta OS = *Homo sapiens* GN = HSP90AB1	1.316	Up
Leukotriene A-4 hydrolase OS = *Homo sapiens* GN = LTA4H	1.264	Up
Heat shock 70 kDa protein 1B OS = *Homo sapiens* GN = HSPA1B	1.67	Up
78 kDa glucose-regulated protein OS = *Homo sapiens* GN = HSPA5	1.266	Up
“Solute carrier family 2, facilitated glucose transporter member 1 OS = *Homo sapiens* GN = SLC2A1”	1.919	Up
“C-1-Tetrahydrofolate synthase, cytoplasmic OS = *Homo sapiens* GN = MTHFD1”	1.365	Up
Alcohol dehydrogenase class-3 OS = *Homo sapiens* GN = ADH5	0.825	Down
Annexin A3 OS = *Homo sapiens* GN = ANXA3	2.256	Up
X-ray repair cross-complementing protein 5 OS = *Homo sapiens* GN = XRCC5	0.777	Down
“HLA class I histocompatibility antigen, A-11 alpha chain OS = *Homo sapiens* GN = HLA-A”	1.296	Up
Endoplasmin OS = *Homo sapiens* GN = HSP90B1	1.214	Up
Bactericidal permeability-increasing protein OS = *Homo sapiens* GN = BPI	1.773	Up
Phosphoglycerate mutase 1 OS = *Homo sapiens* GN = PGAM1	1.257	Up
Nucleolin OS = *Homo sapiens* GN = NCL	0.748	Down
Neutrophil cytosol factor 2 OS = *Homo sapiens* GN = NCF2	1.415	Up
“V-type proton ATPase subunit B, brain isoform OS = *Homo sapiens* GN = ATP6V1B2”	1.262	Up
Nonspecific lipid-transfer protein OS = *Homo sapiens* GN = SCP2	1.359	Up
Nucleoside diphosphate kinase B OS = *Homo sapiens* GN = NME2	1.328	Up
cAMP-dependent protein kinase catalytic subunit beta OS = *Homo sapiens* GN = PRKACB	0.649	Down
Cofilin-1 OS = *Homo sapiens* GN = CFL1	0.77	Down
Cathepsin S OS = *Homo sapiens* GN = CTSS	1.219	Up
Protein S100-P OS = *Homo sapiens* GN = S100P	3.417	Up
Erythrocyte band 7 integral membrane protein OS = *Homo sapiens* GN = STOM	1.392	Up
Calreticulin OS = *Homo sapiens* GN = CALR	1.243	Up
Transketolase OS = *Homo sapiens* GN = TKT	1.392	Up
Flavin reductase (NADPH) OS = *Homo sapiens* GN = BLVRB	1.609	Up
“Peptidyl-prolyl *cis*-trans isomerase F, mitochondrial OS = *Homo sapiens* GN = PPIF”	0.812	Down
Coronin-1A OS = *Homo sapiens* GN = CORO1A	0.822	Down
Protein S100-A11 OS = *Homo sapiens* GN = S100A11	1.698	Up
Peroxiredoxin-2 OS = *Homo sapiens* GN = PRDX2	1.967	Up
Transaldolase OS=*Homo sapiens* GN = TALDO1	1.25	Up
Macrophage-capping protein OS = *Homo sapiens* GN = CAPG	1.339	Up
“Malate dehydrogenase, cytoplasmic OS = *Homo sapiens* GN = MDH1”	0.702	Down
Myeloid cell nuclear differentiation antigen OS = *Homo sapiens* GN = MNDA	1.259	Up
LIM and senescent cell antigen-like-containing domain protein 1 OS = *Homo sapiens* GN = LIMS1	0.799	Down
“6-Phosphogluconate dehydrogenase, decarboxylating OS = *Homo sapiens* GN = PGD”	1.609	Up
Histone H4 OS = *Homo sapiens* GN = HIST1H4A	0.309	Down
Tubulin alpha-4A chain OS = *Homo sapiens* GN = TUBA4A	0.721	Down
Hemoglobin subunit beta OS = *Homo sapiens* GN = HBB	1.475	Up
Neutrophil gelatinase-associated lipocalin OS = *Homo sapiens* GN = LCN2	1.714	Up
“Phosphate carrier protein, mitochondrial OS = *Homo sapiens* GN = SLC25A3”	0.719	Down
14-3-3 protein eta OS = *Homo sapiens* GN = YWHAH	1.271	Up
Selenium-binding protein 1 OS = *Homo sapiens* GN = SELENBP1	1.342	Up
Integrin-linked protein kinase OS = *Homo sapiens* GN = ILK	0.819	Down
Stromal interaction molecule 1 OS = *Homo sapiens* GN = STIM1	0.764	Down
Four and a half LIM domains protein 1 OS = *Homo sapiens* GN = FHL1	0.653	Down
Protein disulfide-isomerase A6 OS = *Homo sapiens* GN = PDIA6	1.223	Up
Septin-7 OS = *Homo sapiens* GN = SEPT7	0.822	Down
Putative elongation factor 1-alpha-like 3 OS = *Homo sapiens* GN = EEF1A1P5	1.222	Up
Phospholipase B-like 1 OS = *Homo sapiens* GN = PLBD1	1.748	Up
Olfactomedin-4 OS = *Homo sapiens* GN = OLFM4	3.945	Up
Fermitin family homolog 3 OS = *Homo sapiens* GN = FERMT3	0.772	Down
Histone H2A type 1-C OS = *Homo sapiens* GN = HIST1H2AC	0.592	Down
Sarcoplasmic/endoplasmic reticulum calcium ATPase 3 OS = *Homo sapiens* GN = ATP2A3	0.833	Down
WW domain-binding protein 2 OS = *Homo sapiens* GN = WBP2	1.24	Up
EF-hand domain-containing protein D2 OS = *Homo sapiens* GN = EFHD2	0.78	Down
Histone H2A type 1-J OS = *Homo sapiens* GN = HIST1H2AJ	0.732	Down
Beta-parvin OS = *Homo sapiens* GN = PARVB	0.815	Down
Alpha-hemoglobin-stabilizing protein OS = *Homo sapiens* GN = AHSP	1.824	Up
Bridging integrator 2 OS = *Homo sapiens* GN = BIN2	0.824	Down
Coronin-1C OS = *Homo sapiens* GN = CORO1C	0.808	Down
Protein kinase C and casein kinase substrate in neurons protein 2 OS = *Homo sapiens* GN = PACSIN2	1.23	Up
Voltage-dependent anion-selective channel protein 3 OS = *Homo sapiens* GN = VDAC3	0.694	Down

The fold change was more than 1.2 times as a significant upregulation and less than 1/1.2 as a criterion for significant downregulation.

## Data Availability

The raw datasets used to support the findings of this study are available from the corresponding author upon request.
